# Effect of DNA markers on the fertility traits of Japanese Black cattle for improving beef quantity and quality

**DOI:** 10.5194/aab-63-9-2020

**Published:** 2020-01-13

**Authors:** Fuki Kawaguchi, Miyako Tsuchimura, Kenji Oyama, Tamako Matsuhashi, Shin Maruyama, Hideyuki Mannen, Shinji Sasazaki

**Affiliations:** 1 Laboratory of Animal Breeding and Genetics, Graduate School of Agricultural Science, Kobe University, Kobe, 657-8501, Japan; 2 Food Resources Education & Research Center, Kobe University, Kasai, 657-2103, Japan; 3 Institute of Advanced Technology, Kindai University, Kinokawa, 649-6493, Japan; 4 Gifu Prefectural Livestock Research Institute, Takayama, 506-0101, Japan

## Abstract

Carcass traits have been efficiently improved by recent selection using DNA markers in beef cattle. Additionally, DNA markers might have an effect on other traits such as fertility traits; therefore attention should also be paid to such pleiotropic effects. However, the effects of the markers on both carcass and fertility traits have never been evaluated in the same population, since they are generally measured in different populations. The objective in the current study was to discuss effectiveness of DNA markers developed for carcass traits through investigation of their effects on carcass and fertility traits in a population. We genotyped six markers *SCD* V293A, *FASN* g.841G>C, *PLAG1* g.49066C>G, *NCAPG* I442M, *DGAT1* K232A, and *EDG1* g.1471620G>T in a Japanese Black cattle population (n=515). To investigate their effects on carcass and fertility traits, we performed statistical analysis (ANOVA and the Tukey–Kramer honestly significant difference (HSD) test). In the results, three of six markers, *SCD* V293A, *NCAPG* I442M, and *EGD1* g.1471620G>T, were significantly associated with both carcass and fertility traits. Remarkably, the same allele for each marker had positive effects on both traits, suggesting that we would be able to simultaneously improve them using these markers in this population. However, previous studies reported that the effects of DNA markers could differ among populations. Therefore, it is necessary to confirm the effect of the marker in each population before it is used for improvement.

## 1 Introduction

DNA markers are a useful tool for genetically improving economic traits in cattle. Several DNA polymorphisms within genes have been identified as DNA markers for improving beef quantity and quality; these include stearoyl-CoA desaturase (*SCD*) V293A (rs41255693), fatty acid synthase (*FASN*) g.841G>C (rs41920005), pleiomorphic adenoma gene 1 (*PLAG1*) g.49066C>G (rs109231213), non-SMC condensin I complex subunit G (*NCAPG*) I442M (rs109570900), diacylglycerol O-acyltransferase 1 (*DGAT1*) K232A (rs109234250), and endothelial differentiation gene 1 (*EDG1*) g.1471620G>T (rs381723690). These are significantly associated with important economic traits in beef cattle, such as fatty acid composition (*SCD* and *FASN*; Taniguchi et al., 2004; Abe et al., 2009; Hayakawa et al., 2015), carcass weight (CW) (*PLAG1* and *NCAPG*; Setoguchi et al., 2009; Nishimura et al., 2012), subcutaneous fat thickness (SFT) (*DGAT1*; Narukami et al., 2011), and beef marbling standard (BMS) (*EDG1*; Yamada et al., 2009). These associations have been confirmed in various cattle populations (Sukegawa et al., 2010; Matsuhashi et al., 2011; Nishimaki et al., 2016; Kim et al., 2017), and the relationship between each trait and gene functions has been explained in previous studies. Therefore, in cattle, these DNA polymorphisms appear to be responsible for carcass traits.

Some markers are also significantly associated with other carcass traits. For instance, *NCAPG* polymorphism is significantly associated with CW, rib eye area (REA), and rib thickness (RT) (Nishimaki et al., 2016). *PLAG1* polymorphism was also significantly associated with CW, REA, SFT, and fatty acid composition (Kigoshi et al., 2018). However, the favorable alleles associated with CW and fatty acid composition differed, suggesting that attention should be paid to the negative effect of DNA polymorphism-based selection on these parameters, although a marker to assess each trait would be useful. Thus, investigating the effects of DNA markers on other economic traits is essential to the consideration of such negative effects.

In the current study, we focused on fertility traits, which have an economic impact that is associated with herd input–output efficiency. Although the heritability of fertility traits is generally low (Kadarmideen et al., 2003; Oyama et al., 2004), genetic factors may contribute to the regulation of fertility traits. In fact, fertility traits unfavorably decline during genetic selection in Holstein populations, suggesting the importance of genetic contribution for fertility traits (Ma et al., 2019). Furthermore, DNA markers for carcass traits have recently been used as indicators for selection. Their effects on fertility traits should be investigated to avoid unconscious decline in fertility traits. However, the effect of DNA markers on both of the traits has never been evaluated in the same population; phenotypic values for carcass and fertility traits have generally been evaluated in different populations, particularly in beef fattening and reproduction cow populations. Thus, the present study aimed to investigate the effect of six DNA markers on both carcass and fertility traits in a Japanese Black cattle population.

## 2 Materials and methods

### 2.1 Animals and traits

We used 515 Japanese Black cows bred from 1990 to 2013 in Gifu Prefecture, Japan. Blood samples were collected from each cow for genomic DNA extraction, which was conducted using the standard phenol–chloroform method (Sambrook and Russell, 2001). All experiments were carried out according to the Kobe University Animal Experimentation Regulations. All blood samples were collected by veterinarians or individual livestock owners. These treatments were carried out in accordance with Japanese Veterinarians Act (Act No. 186 of 1949).

Predicted breeding values for carcass and fertility traits were obtained from the Wagyu Registry Association (Table 1). This association periodically analyzes phenotypic data using models similar to that proposed by Oyama et al. (2004, 2009). Carcass traits considered in this study were CW (kg), REA (cm${}^{2}$), RT (cm), SFT (cm), yield estimate (YE), and BMS; age at first calving (AFC, months), calving interval (CI, days), and the number of calves produced at 4 years of age (${\mathrm{NCP}}_{4}$) were considered as fertility traits. Measurements for these traits were obtained as described by Oyama et al. (2004, 2009).

**Table 1:** Summary statistics of predicted breeding values and heritabilities for carcass and fertility traits in the Japanese Black cattle population (n=515).

	Mean	SD	Min	Max	Heritability
Carcass trait					
CW	464.85	27.48	389.56	569.53	0.496
REA	63.57	4.92	47.81	81.61	0.576
RT	8.40	0.42	7.13	9.80	0.417
SFT	2.34	0.42	1.10	4.00	0.598
YE	75.38	0.86	72.37	78.56	0.639
BMS	3.64	0.55	1.94	5.56	0.608
Fertility trait					
AFC	22.92	0.77	21.09	25.14	0.112
CI	400.91	7.71	377.43	422.82	0.066
${\mathrm{NCP}}_{4}$	2.87	0.05	2.72	3.01	0.093

CW: carcass weight (kg), REA: rib eye area (cm${}^{2}$), RT: rib thickness (cm), SFT: subcutaneous fat thickness (cm), YE: yield estimate (%), BMS: beef marbling standard, AFC: age at first calving (months), CI: calving interval (days), ${\mathrm{NCP}}_{4}$: the number of calves produced at 4 years of age. Heritabilities for carcass and fertility traits were calculated in Japanese Black populations bred in Gifu Prefecture (n=139523) and the whole of Japan (n=1322609–6 730 361).

### 2.2 Genotyping

We genotyped *SCD* V293A, *FASN* g.841G>C, *PLAG1* g.49066C>G, *NCAPG* I442M, *DGAT1* K232A, and *EDG1* g.1471620G>T. We also designed a primer set and two fluorescence-labeled probes to genotype *SCD* polymorphism using the TaqMan single nucleotide polymorphism (SNP) genotyping assay. The following thermocycling conditions were applied: initial denaturation at 95 ${}^{\circ }$C for 10 m at a 10 ${}^{\circ }$C s${}^{-1}$ ramp rate, followed by 40 cycles at 95 ${}^{\circ }$C (4.4 ${}^{\circ }$C s${}^{-1}$ ramp rate) for 10 s, 64 ${}^{\circ }$C (4.0 ${}^{\circ }$C s${}^{-1}$ ramp rate) for 60 s, and 60 ${}^{\circ }$C (1.0 ${}^{\circ }$C s${}^{-1}$ ramp rate) for 30 s. *FASN* and *PLAG1* polymorphisms were also genotyped using the TaqMan SNP genotyping assay, according to method reported by Hayakawa et al. (2015) and Karim et al. (2011), respectively. Polymerase chain reaction and restriction fragment length polymorphism (PCR–RFLP) was applied to genotype *NCAPG*, *DGAT1*, and *EDG1* polymorphisms, as reported by Eberlein et al. (2009), Lacorte et al. (2006), and Yamada et al. (2009), respectively. Primer and probe sequences are shown in Table 2.

**Table 2:** Marker information for genotyping.

Gene	Marker	Position	Target trait	Sequence (5${}^{\prime }$ to 3${}^{\prime }$)	Reference for genotyping
*SCD*	V293A	26: 21144708	FA composition	F: CGC CCT TAT GAC AAG ACC ATC	–
				R: TCT TGC TGT GGA CTG CT	
				fam-CTT ACC CGC AGC TCC CAG G-BHQ	
				hex-ACT TAC CCA CAG CTC CCA GG-BHQ	
*FASN*	g.841G>C	19: 51384984	FA composition	F: ACA CTC CAT CCT CGC TC	Hayakawa et al. (2015)
				R: TCC CGA CTC GCA ACT TC	
				fam-ACA GCC GCC CGC G-BHQ	
				hex-ACA GCC CCC CGC GC-BHQ	
*PLAG1*	g.49066C>G	14: 25003338	CW	F: ATG GGA TCA CCA CAG ACC AT	Karim et al. (2011)
				R: TGC ACA GAA TCA GTG TGT CTT TT	
				fam-ACT TGG GTC AAT ATT T-BHQ	
				hex-CTT GGG TGA ATA TTT-BHQ	
*NCAPG*	I442M	6: 38777311	CW	F: ATT TAG GAA ACG ACT ACT GG	Eberlein et al. (2009)
				R: ATT TGT ATT CTC TTA TTA TCA TC	
*DGAT1*	K232A	14: 1802265	fat thickness	F: GCA CCA TCC TCT TCC TCA AG	Lacorte et al. (2006)
				R: GGA AGC GCT TTC GGA TG	
*EDG1*	g.1471620G>T	3: 42190488	BMS	F: GTG TTA ATA TGT ATG AAG CTT GAT AGT CAG GAA ATA AAT	Yamada et al. (2009)
				R: CCA CTG TAT CGC TGA GCT AGG T	

The positions are given as position in UMD3.1.1.

### 2.3 Statistical analysis

We evaluated the effects of the DNA markers on six carcass and three fertility traits using the generalized least squares method implemented in JMP13 (SAS Institute Inc., Cary, NC, USA). The association was tested using the analysis of variance and the Tukey–Kramer honestly significant difference (HSD) test. All traits were indicated as the sum of overall mean and predicted breeding value.

## 3 Results and discussion

### 3.1 Allele frequencies

We genotyped six DNA markers in a Japanese Black cattle population (n=515). Minor allele frequencies were 0.372 for *SCD* V293A, 0.083 for *FASN* g.841G>C, 0.274 for *PLAG1* g.49066C>G, 0.196 for *NCAPG* I442M, 0.234 for *DGAT1* K232A, and 0.487 for *EDG1* g.1471620G>T (Table 3). These were similar to those reported for Japanese Black cattle (Taniguchi et al., 2004; Yamada et al., 2009; Narukami et al., 2011; Nishimura et al., 2012; Hayakawa et al., 2015).

**Table 3:** Genotype and allele frequency of each marker in the Japanese Black cattle population (n=515).

Marker	Genotype			Allele	
	frequency (n)			frequency	
*SCD*	AA	AV	VV	A	V
	0.396	0.464	0.140	0.628	0.372
	(204)	(239)	(72)		
*FASN*	GG	GC	CC	G	C
	0.843	0.149	0.008	0.917	0.083
	(434)	(77)	(4)		
*PLAG1*	QQ	Qq	qq	Q	q
	0.522	0.408	0.070	0.726	0.274
	(269)	(210)	(36)		
*NCAPG*	II	IM	MM	I	M
	0.641	0.326	0.033	0.804	0.196
	(330)	(168)	(17)		
*DGAT1*	KK	KA	AA	K	A
	0.588	0.355	0.056	0.766	0.234
	(303)	(183)	(29)		
*EDG1*	TT	TG	GG	T	G
	0.254	0.517	0.229	0.513	0.487
	(131)	(266)	(118)		

### 3.2 Effects of six markers on carcass and fertility traits

We investigated the association of six DNA polymorphisms with carcass and fertility traits using statistical analysis. The results indicated that three DNA polymorphisms (*SCD*, *NCAPG*, and *EDG1*) were significantly associated with carcass and fertility traits (Table 4). The rest (*FASN*, *PLAG1*, and *DGAT1*) showed significant association only with carcass traits. Significant differences were also noted between genotypes using least square means (Tables 5, 6). In the following paragraphs, we discuss the effects of each DNA polymorphism on traits and their effectiveness as markers.

**Table 4:** Association between markers and traits in the Japanese Black cattle population (n=515).

	*SCD*	*FASN*	*PLAG1*	*NCAPG*	*DGAT1*	*EDG1*
Carcass trait						
CW	ns	ns	${}^{***}$	${}^{***}$	${}^{***}$	ns
REA	ns	ns	ns	${}^{**}$	ns	${}^{*}$
RT	${}^{***}$	ns	ns	${}^{***}$	${}^{**}$	${}^{**}$
SFT	ns	ns	ns	${}^{*}$	ns	ns
YE	ns	ns	ns	${}^{*}$	ns	ns
BMS	${}^{***}$	${}^{*}$	ns	${}^{*}$	ns	${}^{***}$
Fertility trait						
AFC	${}^{***}$	ns	ns	${}^{***}$	ns	${}^{*}$
CI	ns	ns	ns	ns	ns	ns
${\mathrm{NCP}}_{4}$	${}^{*}$	ns	ns	${}^{***}$	ns	ns

${}^{*}$ p<0.05, ${}^{**}$ p<0.01, ${}^{***}$ p<0.001, ns: nonsignificant.

#### 3.2.1 *SCD* V293A

*SCD* encodes an enzyme involved in fatty acid desaturation. Within this gene, Taniguchi et al. (2004) identified an amino acid substitution (V293A) associated with fatty acid composition in Japanese Black cattle. Moreover, a positive effect of the A allele on fatty acid composition has been confirmed in various cattle populations (Ohsaki et al., 2009; Li et al., 2012; Kim et al., 2017).

Furthermore, some researchers have investigated the effect of *SCD* V293A on other carcass traits. Jomane et al. (2017) reported that *SCD* V293A was significantly associated with RT, YE, and BMS. They suggested that it also affects these traits because they are integrally related to fat. Conversely, Ohsaki et al. (2009) and Matsuhashi et al. (2011) did not observed any association between *SCD* V293A and carcass traits, including CW, REA, RT, SFT, YE, and BMS. In the present study, *SCD * V293A was significantly associated with RT and BMS. Animals with the AA genotype showed significantly larger RT and BMS than those with the AV genotype in the current study. However, the opposite effect was observed in the previous study (Jomane et al., 2017); significantly larger RT and BMS were observed in animals with the AV genotype than in those with the AA genotype. These results suggested that the effect of *SCD* V293A on carcass traits, except for fatty acid composition, may be because of linkage disequilibrium (LD) with a polymorphism responsible for each trait.

With regard to fertility traits, *SCD* V293A was significantly associated with AFC. The Tukey–Kramer HSD test revealed that animals with the AA genotype were of significantly younger AFC than those with the AV and VV genotypes. Asadollahpour Nanaei et al. (2014) were the first to report on the effect of *SCD* polymorphism on fertility traits. They found an association between *SCD* V293A and two fertility traits, pregnancy length and open days, in Iranian Holstein cattle. They concluded that SNP may indirectly affect fertility traits, as the precise molecular mechanism underlying the reported effect could not be determined. However, the novel role of *SCD* in ovaries was recently elucidated. *SCD* maintains the developmental competence of oocytes by reducing the amount of saturated fatty acids, which induces lipotoxicity (Aardema et al., 2017). V293A may accordingly have a similar effect on fertility traits such as AFC and open days through the regulation of ovulation. In conclusion, *SCD* V293A may be a useful DNA marker of both AFC and fatty acid composition, as the selection of the A allele results in the reduction of AFC and the improvement of fatty acid composition.

#### 3.2.2 *FASN* g.841G>C

Some polymorphisms within *FASN* have been reported to be significantly associated with fatty acid composition. Abe et al. (2009) determined an association between fatty acid composition and two *FASN* amino acid substitutions, g.16024A>G (T1952A) and g.16039T>C (W1957R), which were in complete LD. Although their effect on other carcass traits has been investigated, a significant association has not been observed (Matsuhashi et al., 2011; Nishimaki et al., 2016; Kim et al., 2017). Hayakawa et al. (2015) also identified the polymorphism *FASN* g.841G>C to be associated with fatty acid composition within the gene promoter. They used the same population as was used in the current study and demonstrated that g.841G>C had a stronger effect on fatty acid composition than T1952A or W1957R. Therefore, we selected *FASN* g.841G>C for assessment in the current study.

In this study, we investigated the effect of *FASN* g.841G>C on six carcass traits and three fertility traits and found that it was significantly associated with BMS. The selection of the G allele may improve BMS, as the GG genotype showed higher BMS than the GC genotype. Furthermore, the G allele increases the amount of unsaturated fatty acids (Hayakawa et al., 2015). These results suggest that *FASN* g.841G>C is an effective DNA marker for improving beef quality.

#### 3.2.3 *PLAG1* g.49066C>G

*PLAG1* regulates gene expression related to cattle growth via transcription factors such as *insulin-like growth factor 2* (*IGF-2*) (Voz et al., 2004). In the current study, *PLAG1* g.49066C>G was significantly associated with CW, and animals with the QQ genotype showed significantly higher CW than those with the Qq and qq genotypes, which is consistent with the findings of previous reports (Karim et al., 2011; Nishimura et al., 2012).

In this study, ANOVA revealed that *PLAG1* g.49066C>G was not significantly associated with any traits except for CW (Table 4), although the significant difference between QQ and Qq genotypes was detected for CI in the Tukey–Kramer HSD test (Table 5). These results suggested that we would not have to pay attention to the effect on any other traits, since the marker would have little effect on them. However, the marker has been reported to be significantly associated with REA (Hoshiba et al., 2013) and fatty acid composition (Kigoshi et al., 2018). Kigoshi et al. (2018) reported that the Q allele significantly reduced oleic acid percentage compared with the q allele. Although this polymorphism would be a useful DNA marker for improving CW without negative effects on other traits investigated in the current study, further study on the effect of this DNA marker on fatty acid composition is required to precisely evaluate its availability as a DNA marker for selection.

**Table 5:** The effect of the markers within *SCD*, *FASN*, and *PLAG1* genes on carcass and fertility traits in the Japanese Black cattle population (n=515).

	*SCD*			*FASN*			*PLAG1*		
	AA (204)	AV (239)	VV (72)	GG (434)	GC (77)	CC (4)	QQ (269)	Qq (210)	qq (36)
Carcass trait									
CW	**468.3**${}^{a}$	**462.1**${}^{b}$	**464.0**${}^{\mathrm{ab}}$	464.7	465.1	471.0	**470.2**${}^{a}$	**460.2**${}^{b}$	**452.1**${}^{b}$
	1.92	1.77	3.23	1.32	3.14	13.76	1.64	1.86	4.48
REA	63.97	63.42	62.91	63.61	63.30	63.75	64.04	63.14	62.50
	0.34	0.32	0.58	0.24	0.56	2.46	0.30	0.34	0.82
RT	**8.49**${}^{a}$	**8.35**${}^{b}$	**8.30**${}^{b}$	8.40	8.38	8.70	8.40	8.41	8.31
	0.03	0.03	0.05	0.02	0.05	0.21	0.03	0.03	0.07
SFT	2.34	2.32	2.40	2.34	2.37	2.10	2.33	2.35	2.35
	0.03	0.03	0.05	0.02	0.05	0.21	0.03	0.03	0.07
YE	75.45	75.38	75.18	75.39	75.30	75.81	75.38	75.38	75.33
	0.06	0.06	0.10	0.04	0.10	0.43	0.05	0.06	0.14
BMS	**3.79**${}^{a}$	**3.59**${}^{b}$	**3.36**${}^{c}$	**3.66**${}^{a}$	**3.48**${}^{b}$	**3.45**${}^{\mathrm{ab}}$	3.67	3.61	3.47
	0.04	0.03	0.06	0.03	0.06	0.27	0.03	0.04	0.09
Fertility trait									
AFC	**22.76**${}^{b}$	**22.99**${}^{a}$	**23.16**${}^{a}$	22.89	23.09	22.85	22.88	22.94	23.19
	0.05	0.05	0.09	0.04	0.09	0.39	0.05	0.05	0.13
CI	400.1	401.5	401.2	400.8	401.7	401.2	**401.6**${}^{a}$	**399.9**${}^{b}$	**401.3**${}^{\mathrm{ab}}$
	0.54	0.50	0.91	0.37	0.88	3.86	0.47	0.53	1.28
${\mathrm{NCP}}_{4}$	2.88	2.87	2.86	2.87	2.87	2.88	2.87	2.87	2.85
	0.004	0.003	0.006	0.002	0.006	0.026	0.003	0.004	0.009

Values represent the least square means (upper row) and SE (lower row) for each genotype. Means with different superscripts (a, b) are significantly different between genotypes. Bold type represents significant differences (p<0.05).

**Table 6:** The effect of the markers within *NCAPG*, *DGAT1*, and *EDG1* genes on carcass and fertility traits in the Japanese Black cattle population (n=515).

	*NCAPG*			*DGAT1*			*EDG1*		
	II (330)	IM (168)	MM (17)	KK (303)	KA (183)	AA (29)	TT (131)	TG (266)	GG (118)
Carcass trait									
CW	**460.2**${}^{b}$	**472.2**${}^{a}$	**482.1**${}^{a}$	**460.3**${}^{b}$	**471.1**${}^{a}$	**472.5**${}^{\mathrm{ab}}$	468.9	464.5	461.2
	1.47	2.07	6.49	1.55	2.00	5.01	2.39	1.68	2.52
REA	**63.04**${}^{b}$	**64.44**${}^{a}$	**65.12**${}^{\mathrm{ab}}$	63.30	63.80	64.83	**64.61**${}^{a}$	**63.36**${}^{b}$	**62.87**${}^{b}$
	0.27	0.38	1.18	0.28	0.36	0.91	0.43	0.30	0.45
RT	**8.34**${}^{b}$	**8.49**${}^{a}$	**8.52**${}^{\mathrm{ab}}$	**8.35**${}^{b}$	**8.44**${}^{\mathrm{ab}}$	**8.55**${}^{a}$	**8.48**${}^{a}$	**8.40**${}^{a}$	**8.29**${}^{b}$
	0.02	0.03	0.10	0.02	0.03	0.08	0.04	0.03	0.04
SFT	2.37	2.29	2.22	2.34	2.35	2.35	2.39	2.33	2.30
	0.02	0.03	0.10	0.02	0.03	0.08	0.04	0.03	0.04
YE	**75.30**${}^{b}$	**75.51**${}^{a}$	**75.58**${}^{\mathrm{ab}}$	75.37	75.36	75.54	75.47	75.37	75.30
	0.05	0.07	0.21	0.05	0.06	0.16	0.07	0.05	0.08
BMS	**3.59**${}^{b}$	**3.74**${}^{a}$	**3.55**${}^{\mathrm{ab}}$	3.62	3.64	3.75	**3.83**${}^{a}$	**3.62**${}^{b}$	**3.46**${}^{c}$
	0.03	0.04	0.13	0.03	0.04	0.10	0.05	0.03	0.05
Fertility trait									
AFC	**23.11**${}^{a}$	**22.59**${}^{b}$	**22.63**${}^{b}$	22.92	22.97	22.67	**22.83**${}^{b}$	**22.89**${}^{b}$	**23.10**${}^{a}$
	0.04	0.06	0.18	0.04	0.06	0.14	0.07	0.05	0.07
CI	401.4	400.4	397.3	400.3	401.8	401.3	**402.3**${}^{a}$	**400.4**${}^{b}$	**400.6**${}^{\mathrm{ab}}$
	0.42	0.59	1.86	0.44	0.57	1.43	0.67	0.47	0.70
${\mathrm{NCP}}_{4}$	**2.86**${}^{b}$	**2.89**${}^{a}$	**2.91**${}^{a}$	2.87	2.87	2.88	2.87	2.88	2.87
	0.003	0.004	0.012	0.003	0.004	0.010	0.005	0.003	0.005

Values represent the least square means (upper row) and SE (lower row) for each genotype. Means with different superscripts (a,b) are significantly different between genotypes. Bold type represents significant differences (p<0.05).

#### 3.2.4 *NCAPG* I442M

*NCAPG *I442M has also been regarded as a DNA marker that is strongly associated with CW. As this gene encodes a condensin subunit that plays an important role in cell proliferation (Dej et al., 2004), it may be responsible for cattle growth (Setoguchi et al., 2009). We observed a significantly higher CW in animals with the M allele in the DNA marker, as demonstrated by previous studies (Lindholm-Perry et al., 2011; Hoshiba et al., 2013; Nishimaki et al., 2016). In addition, this marker was significantly associated with all other carcass traits investigated in the current study. Moreover, *NCAPG* I442M is significantly associated with REA (Hoshiba et al., 2013; Nishimaki et al., 2016), RT (Nishimaki et al., 2016), SFT (Setoguchi et al., 2009; Hoshiba et al., 2013), and YE (Hoshiba et al., 2013) in Japanese Black cattle. In these studies, REA, RT, and YE increased in animals with the M allele while SFT increased in those with the I allele. As our results were consistent with those of previous studies, the effect of *NCAPG* I442M on carcass traits may be common in Japanese Black cattle populations.

In a previous study, positive genetic correlations were detected among CW, REA, RT, YE, and BMS in Japanese Black cattle (Oyama, 2011). Genetic correlation would be generally caused by effects of a polymorphism directly influencing both traits or linkage disequilibrium between responsible polymorphisms. Considering the function of the *NCAPG* gene in cell proliferation and associations observed in various populations, the marker in the *NCAPG* gene might affect all carcass traits. Therefore, the effect of the polymorphism on these carcass traits might be one of the factors for the genetic correlations.

*NCAPG* I442M was also significantly associated with AFC and ${\mathrm{NCP}}_{4}$ in the current study. Some previous studies have reported genetic correlations between AFC and some growth-related traits. Grossi et al. (2009) reported that AFC favorably correlated with body weight at 365 and 450 d of age (-0.38 and -0.33, respectively). Krpálková et al. (2014) also reported a favorable correlation between AFC and the average daily weight gain (-0.34). Thus, fast-growing cows could calve at an early age. The results of the present study are consistent with those of these previous reports; the M allele of *NCAPG* I442M favorably affected CW and AFC. Therefore, the association between AFC and *NCAPG* I442M may have resulted from the effect of polymorphism through the regulation of cattle growth. In conclusion, the M allele of *NCAPG* has a positive impact on CW, BMS, and AFC, suggesting that the selection of this allele results in improvement of these traits.

#### 3.2.5 *DGAT1* K232A

*DGAT1* K232A has been reported to affect fat percentage and fatty acid composition in milk (Grisart et al., 2004; Schennink et al., 2007). Other studies have reported its significant association with intramuscular fat content (Thaller et al., 2003) and SFT (Narukami et al., 2011) in beef cattle. According to these reports, animals with the K allele showed greater fat content in milk and lower fat content in beef. Narukami et al. (2011) suggested that the role of *DGAT1* in mammary glands is different from that in muscle tissue even though the underlying molecular mechanism remains unknown. In the present study, we observed that *DGAT1* K232A was significantly associated with CW and RT. Considering that intramuscular fat content, SFT, CW, and RT are related to fat content, *DGAT1* K232A or another gene in LD with *DGAT1* may be responsible for fat content as well. However, the effects of *DGAT1* K232A differed among animals, and we therefore need to investigate and consider the effect of this marker on fat-related traits in each animal.

#### 3.2.6 *EDG1* g.1471620G>T

Yamada et al. (2009) identified an SNP (g.1471620G>T) associated with BMS within *EDG1*. They reported that this SNP may possibly be the polymorphism responsible for BMS in terms of gene location and function. The association between this marker and BMS has been confirmed in some populations (Sukegawa et al., 2010; Watanabe et al., 2010). Previous studies have also reported that animals with the T allele showed higher BMS than those with the G allele. In this study, we also observed a significant association between this marker and BMS, with a positive impact of the T allele on that trait. In addition, *EDG1* g.1471620G>T was significantly associated with REA and RT, which was also observed in a previous study (Sukegawa et al., 2010). This marker can be used to improve BMS as well as REA and RT.

To the best of our knowledge, this is the first study to investigate the effect of *EDG1* polymorphism on fertility traits. We found that this marker was significantly associated with AFC. Specifically, *EDG1*, also known as *S1P receptor 1* (*S1P*${}_{1})$, is involved in angiogenesis (Liu et al., 2000). Recently, the family of S1P receptors has been reported to play an important role in ovarian angiogenesis (Von Otte et al., 2006). In addition, *EDG1* expression has been observed in ovarian tissues (Kon et al., 1999), suggesting that the *EDG1* signal may regulate ovarian angiogenesis. Generally, ovarian angiogenesis seems to be one of the factors responsible for follicular development (Geva et al., 2000; Di Pietro et al., 2016). The effect of *EDG1* g.1471620G>T on AFC could be explained by follicular development occurring at different rates due to angiogenesis between genotypes. Hence, *EDG1* g.1471620G>T could be a useful DNA marker of BMS and AFC.

On the other hand, *EDG1 *polymorphism was not significantly associated with CI even though the moderate genetic correlation was previously detected between AFC and CI in Japanese Black cattle (Oyama et al., 2002). The genetic correlation between AFC and CI has been investigated in various breeds (Frazier et al., 1999; Haile-Mariam and Kassa-Mersha, 1994; Ojango and Pollott, 2001). In these previous reports, the correlation was largely varied between breeds (from -0.93 to 0.89), suggesting that the correlation might be caused by linkage disequilibrium between responsible polymorphisms for AFC and CI. In the population used in the current study, *EDG1* polymorphism might not be in linkage disequilibrium with a responsible polymorphism for CI, although it might be responsible for AFC. In conclusion, we would be able to improve AFC without effect on CI using this marker.

## 4 Conclusions

This was the first study to evaluate the effect of six DNA markers on carcass and fertility traits in one population. Three of these DNA markers, *SCD* V293A, *NCAPG* I442M, and *EGD1* g.1471620G>T, were significantly associated with both carcass and fertility traits. Remarkably, statistical analysis revealed that the same allele for each marker had positive effects on both the traits. These results suggest that we can simultaneously improve these traits using the aforementioned DNA markers in this population. However, the effects of DNA markers could differ among populations. Therefore, it is necessary to confirm the effect of the marker in each population before their use.

## Data Availability

The sample data were collected by Tamako Matsuhashi and Shin Maruyama in Gifu Prefecture. The data were analyzed and provided for only this work through Wagyu Registry Association. Therefore, the first and corresponding authors have no right to share the data.
